# Emergence of Azithromycin-resistant Salmonella Typhi in Southeast Asia: A systematic review

**DOI:** 10.12669/pjms.41.7.12194

**Published:** 2025-07

**Authors:** Sehrish Zaffar, Ayesha Sadiqa

**Affiliations:** 1 Sehrish Zaffar, MBBS, MPhil, CHPE, FCPS-Pharmacology, Associate Professor, Pharmacology Department Services Institute of Medical Sciences, Lahore - Pakistan.; 2 Ayesha Sadiqa, BDS, M.Phil. Physiology, CHPE, Ph.D. Physiology, MCPS-HPE (cont.) Associate Professor of Physiology at Physiology Department, CMH Lahore Medical College and Institute of Dentistry, Abdur Rahman Road, Lahore Cantt, Lahore, Punjab, Pakistan

**Keywords:** Bacterial, Drug Resistance, Microbial, Sanitation, Typhoid Fever, Vaccines, Water Supply

## Abstract

**Objective:**

To systematically review the literature on the epidemiology, antimicrobial resistance, and control strategies for typhoid fever, focusing on Southeast Asia, where the disease burden and emergence of drug-resistant strains are significant.

**Method:**

A systematic review was used, and twenty-five manuscripts fulfilling the inclusion criteria were included. Only human studies affected by typhoid fever were included, including systematic reviews, narrative reviews, meta-analyses, cross-sectional studies, original studies, editorials, or Commentary. Manuscripts were searched and reviewed according to PRISMA guidelines. From 2006 to 2024, six reliable sources, including PubMed, Web of Science, Scopus, Cochrane Library, Google Scholar, and Embase, were evaluated.

**Results:**

Typhoid fever remains a significant global health burden, particularly in regions with poor sanitation. In 2019, there were about 9.2 million cases and 110,000 deaths, with the highest rates in the Eastern Mediterranean, Africa, and Southeast Asia. Treatment options exist, and the emergence of drug-resistant strains, including those resistant to first-line antibiotics like azithromycin, poses a serious threat. The misuse of antibiotics primarily causes this resistance. Effective control strategies require improved sanitation and hygiene, widespread vaccination, prudent antibiotic use and stewardship programs, enhanced surveillance, and drug resistance monitoring.

**Conclusion:**

Typhoid, caused by Salmonella typhi, remains a health threat in Southeast Asia due to poor sanitation and limited access to clean water. Addressing this requires improved sanitation, vaccination, careful antibiotic use, and intense surveillance to combat antibiotic-resistant strains.

## INTRODUCTION

Typhoid fever is a bacterial infection caused by *Salmonella Typhi (S. Typhi)*. S. Typhi is a gram-negative rod primarily transmitted through consuming contaminated food or water. Typhoid remains a significant public health concern, especially in regions with poor sanitation and limited access to clean water.^1^ There were 9.2 million cases of typhoid and 110,000 deaths reported globally in 2019. The Eastern Mediterranean (187 cases per 100,000 people), Africa (111 cases), and Southeast Asia (306 cases per 100,000 people) regions had the highest estimated incidences^2^ between 2016 and 2019, adjusted incidence rates in two healthcare facilities in Pakistan were 176 and 103 per 100,000 individuals.^3^

A persistent, high fever marks typhoid fever and may include a rose-colored rash, headaches, weakness, and abdominal pain. Gastrointestinal symptoms like constipation or diarrhea can also occur. The incubation period is typically one to three weeks. Some individuals may carry the bacteria without symptoms, and severe complications can include intestinal perforation, gastrointestinal bleeding, and, in rare cases, death.^4^ Antibiotic therapies are the mainstay of treatment for typhoid fever. This is supplemented with antipyretics and adequate hydration. Most of the patients respond well to oral therapy. However, patients with severe gastrointestinal symptoms often require hospitalization and intravenous drug administration.^5^

Chloramphenicol remained the drug of choice against typhoid fever for many years, but the appearance of serious side effects and the emergence of resistance caused a decline in its use. In 1970, cotrimoxazole (trimethoprim-sulfamethoxazole) and ampicillin became the first-line drugs for managing typhoid fever. However, the development of plasmid-mediated resistance led to the emergence of multi-drug-resistant (MDR) typhoid fever that became unresponsive to ampicillin, chloramphenicol, and co-trimoxazole.^6^ Third-generation cephalosporins and fluoroquinolones have effectively managed the disease in the past decade. Oral options like cefixime and ciprofloxacin are commonly prescribed, while intravenous ceftriaxone and ciprofloxacin are used for parenteral treatment. Unfortunately, the misuse of these antibiotics has led to resistant strains of S. Typhi, resulting in therapeutic failures.^7^

Azithromycin is the first-line treatment option against multidrug-resistant typhoid fever, which is standard in endemic areas such as Southeast Asia and Pakistan. Carbapenems and aztreonam are reserved for patients who fail to respond to ceftriaxone and azithromycin.^8^ The current study aimed to systematically review the literature on the epidemiology, antimicrobial resistance, and control strategies for typhoid fever, focusing specifically on Southeast Asia. The disease’s burden and the emergence of drug-resistant strains present significant public health challenges. The review analyzed various studies to provide a comprehensive understanding of the current situation and effective measures to combat this health threat in the region.

## METHODS

**Fig.1:**
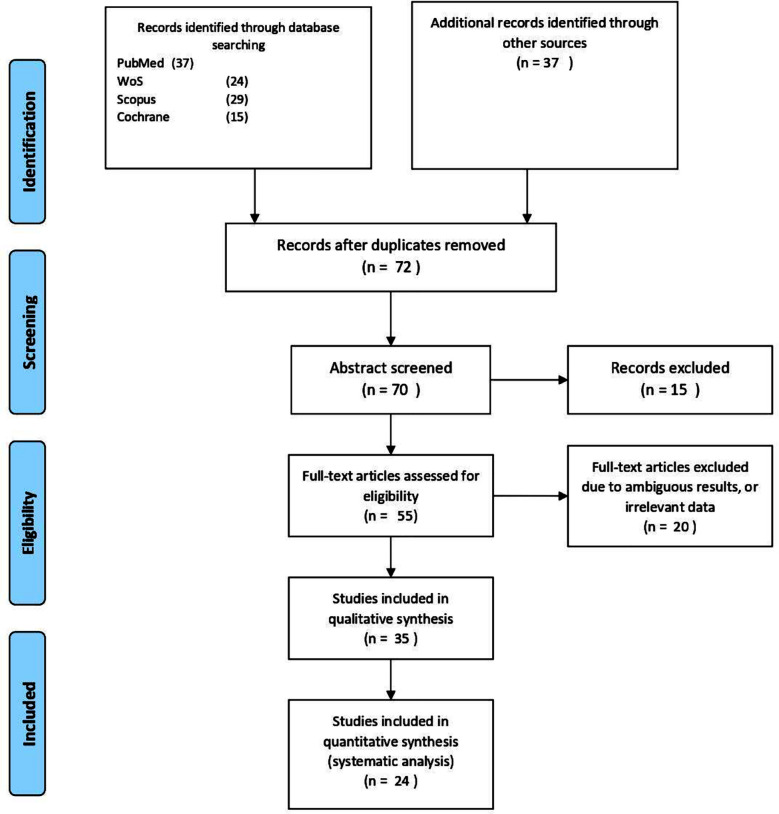
The PRISMA diagram representing the systematic review flow of information.

The present systematic review adheres to the Preferred Reporting Items for Systematic Reviews and Meta-Analyses (PRISMA) (Fig.1).

### Review Questions:

The review questions are:

1. What is the current burden of typhoid fever in Southeast Asia, focusing on drug-resistant strains?
2. Has typhoid fever’s epidemiology shifted over time in Southeast Asia regarding drug resistance?
3. What are the current treatment options for typhoid fever in the endemic regions, and how well do they address antibiotic-drug resistance?
4. What role does vaccination play in controlling typhoid fever in the context of antibiotic-drug resistance in Southeast Asia?
5. What public health interventions most effectively prevent typhoid fever and address antibiotic-drug resistance in Southeast Asia?

### Literature Sources and Inclusion Criteria:

Thorough search engines and indices, including PubMed, Web of Science, Scopus, Cochrane Library, Google Scholar, and Embase, did this systematic review. All the studies included in the review were from 2006 to 2024. The keywords used to search the relevant literature were bacteria, drug resistance, microbial, vaccines, sanitation, typhoid fever, and water supply. All studies were thoroughly reviewed to meet established inclusion and exclusion criteria, focusing on individual manuscript components, particularly the titles, abstracts, methods, results, and conclusions.

- Only human studies affected by Salmonella typhoid fever were included
- All the studies were in the medium of English
- Studies showing the results related to the emergence of Azithromycin-resistant Salmonella Typhi, Multi-drug resistance (MDR), and extensively-drug-resistant (XDR) typhoid were includeds
- All the studies comprised data from Southeast Asia regions.
- The studies can be in the form of systematic reviews, narrative reviews, meta-analyses, cross-sectional studies, original studies, editorials, or Commentary.

### Screening Process for Quality Assessment:

After screening, a quality assessment was conducted to eliminate reporting biases caused by differences in methodology and presentation of results across various populations. This systematic review included twenty four manuscripts that met the inclusion criteria. Each manuscript was evaluated for its relevance, reliability, and overall contribution to understanding the epidemiology and antimicrobial resistance associated with typhoid fever in Southeast Asia. By ensuring high-quality studies were included, the review aimed to provide a comprehensive overview of the current knowledge regarding typhoid fever and the emergence of azithromycin-resistant strains. This process was essential in drawing meaningful conclusions and making informed recommendations for public health interventions and research priorities.

## RESULTS

Typhoid fever is a leading cause of illness and death in Southeast Asia and poses a growing threat due to antimicrobial resistance. The emergence of MDR and XDR S. Typhi, resistant to key antibiotics like ciprofloxacin and ceftriaxone, raises significant concerns. Azithromycin-resistant strains in South Asia, especially in Pakistan, Nepal, and India, further limit treatment options. Global travel also facilitates the spread of these resistant strains, highlighting the need for international surveillance. The high disease burden in South Asia, especially among children, is mainly due to limited access to clean water and sanitation. This situation, combined with inappropriate antibiotic use and self-medication, worsens antibiotic resistance and public health issues. The rise of XDR-typhoid necessitates less desirable treatments like carbapenems. Accurate diagnosis and susceptibility testing are crucial for effective management. Vaccination with typhoid conjugate vaccines is vital in endemic regions. Improved sanitation and hygiene are essential for disease prevention, and antibiotic stewardship programs are needed to combat resistance.

**Fig.2:**
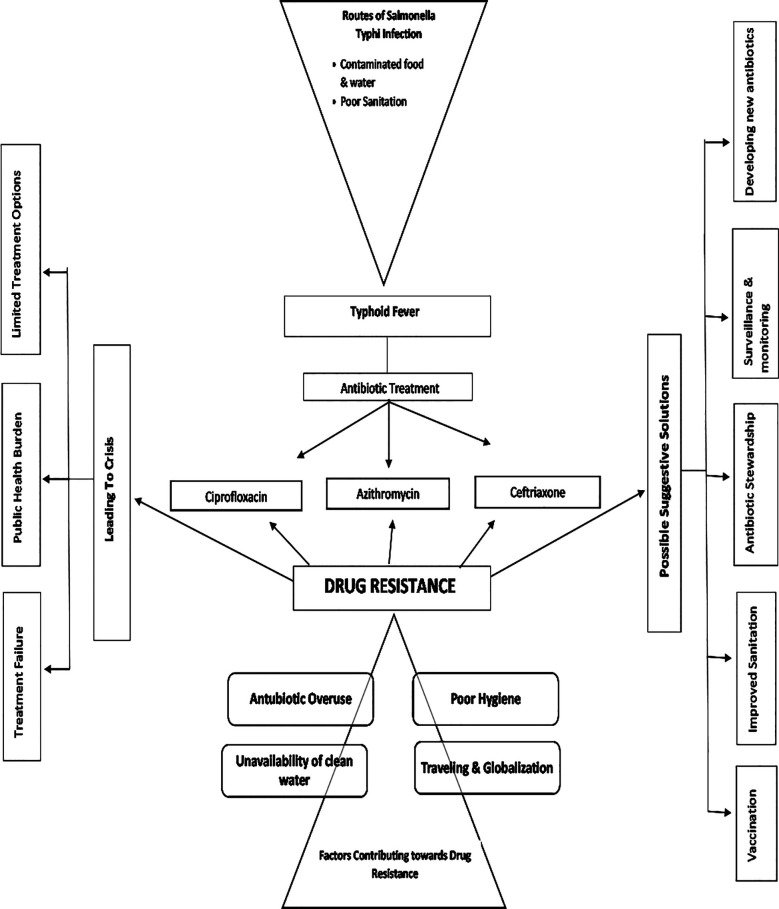
Concerns of Antibiotic drug-resistant S. Typhi and its possible solutions.

The emergence of azithromycin resistance necessitates the search for new therapeutic options. The development of rapid diagnostic tests for drug-resistant strains is crucial for guiding treatment decisions. Surveillance of antibiotic resistance patterns is vital to inform public health interventions. This body of research underscores the urgent need for a multi-pronged approach to combating drug-resistant typhoid, including improved sanitation, vaccination programs, and the judicious use of antibiotics (Table-I, Fig.2).

**Table-I:** Brief overview of referenced literature.

Author	Publication year	Country	Study design	Study Conclusion
Masuet-Aumatell C, Atouguia J.	2021	Worldwide	Narrative review	The rise of drug-resistant typhoid strains mainly affects travelers in endemic areas. Vaccination is advised to prevent typhoid fever, particularly along high-incidence routes.
M Hancuh et al	2023	Worldwide	Population-based world-wide report	Since 2019, Southeast Asia has reported the highest incidence of typhoid, with 306 cases per 100,000 people, mainly in the Eastern Mediterranean. These outbreaks have promoted the use of typhoid vaccines.
Garrett DO et al	2022	Bangladesh, Nepal, and Pakistan	Hybrid surveillance model for incidence estimation of enteric fever	In South-Asian countries, the incidence of enteric fever is very high, with children at the most significant risk. Preventive measures include enhancing water and sanitation infrastructure and typhoid vaccinations.
Manesh A et al	2021	Indian subcontinent	Review article	Despite a global decline in enteric fever, the Indian subcontinent still faces high rates. Antimicrobial resistance restricts treatment options, with ceftriaxone and azithromycin commonly used, even as highly resistant typhoid strains appear in Pakistan. Safe food and water offer limited prevention, and vaccination provides safety for travelers.
Bhutta ZA.	2006	Pakistan	Clinical review	Typhoid fever remains a cause of illness in developing countries, particularly among young children, due to contaminated food and water. Increasing drug resistance to first-line antibiotics complicates management. Prompt diagnosis is crucial for public health, yet vaccines are not widely used for children.
Butler T	2011	South-Asian countries	Review based on clinical trials	Azithromycin as an effective 5-day treatment. Fluoroquinolones are more effective than cephalosporins, with gatifloxacin outperforming ciprofloxacin and ofloxacin in cases of reduced susceptibility to ciprofloxacin. Despite potential bone marrow toxicity and resistance, ceftriaxone and chloramphenicol are still good treatment options in developing countries.
Walker J, et al.	2023	Pakistan	Data analysis taken from International Air Transport Association (IATA)	S. Typhi (H58-haplotype) is closely linked to air travel frequency. Outbreaks of extensively-drug-resistant (XDR) typhoid are found in countries like Pakistan, where local transmission is likely.
Qureshi S et al	2020	Pakistan	Retrospective review	Treatment with azithromycin and meropenem alone or in combination showed similar cure times for XDR-Typhoid. However, azithromycin’s lower cost and oral intake route make it preferable in lower socio-economic countries.
Jabeen K et al	2023	Pakistan	Retrospective cross-sectional study	Disk diffusion and E-tests are unreliable for determining azithromycin resistance in S. Typhi compared to broth microdilution (BMD), leading to a decline in azithromycin use for XDR S. BMD is the most reliable method for assessing azithromycin sensitivity, and sensitivity patterns should be reported with the minimum inhibitory concentration (MIC).
Iqbal J. et al	2020	Nepal, India, Bangladesh, Afghanistan, Pakistan, Iraq, South Africa, Asia, Palestine	Observational study	The rise of XDR-Typhi in Pakistan has made azithromycin the only effective oral treatment. This highlights the urgent need for a typhoid conjugate vaccine in endemic areas.
Jabeen K et al	2023	Pakistan	Cross-sectional descriptive study	Extensively drug-resistant XDR isolates of S. Typhi in Pakistan have developed resistance to first-line and second-line antibiotics and CTX-M genes, diminishing their susceptibility to third-generation cephalosporins. Resistance to azithromycin, an empirical treatment option, is also a concern and requires careful monitoring in endemic areas.
Ahmad S et al	2021	Pakistan	Commentary	Poor sanitation during the COVID-19 pandemic has led to a rise in typhoid cases, while the overuse of azithromycin may hinder treatments for XDR infections.
Fida S et al	2021	Pakistan	Cross-sectional observational study	The growing resistance to typhoid highlights the need for prevention, effective infection management, and prudent antibiotic use in endemic countries like Pakistan.
Aziz S, and Malik L.	2018	Pakistan	Short communication	To control Multi-drug resistance (MDR) enteric fever in Pakistan, measures include mass immunization (with attenuated Typhi 21a or injectable unconjugated Vi typhoid), rational antibiotic prescription, the availability of clean public sanitation and pure drinking water, safe food, and public health education.
Sajib MSI et al	2021	Bangladesh	Data reporting based on DNA extraction and whole-genome sequencing	Typhoid/paratyphoid fever is widespread in South Asia and is treated with antimicrobial drugs. In Pakistan, antimicrobial resistance has surged since 2016, when extensively drug-resistant Typhi left azithromycin as the sole oral option. This has been indicated by a mutation in acrB (AcrB-R717Q/L) since 2019. Low-cost rapid PCR for detecting pan-oral drug-resistant S. Typhi has been suggested.
Ahsan S, and Rahman S.	2019	Bangladesh	Original article	Macrolide resistance in Salmonella enterica serovars Typhi and Paratyphi is a primary concern in Bangladesh, where azithromycin is widely used for treatment.
Duy PT et al	2020	Nepal	Original study	This is the first molecular report of azithromycin-resistant S. Typhi in Nepal. These strains show no genetic link to those in Bangladesh, indicating that rising azithromycin use may drive the emergence of resistance in South Asia.
Carey ME et al	2021	Bangladesh, Pakistan, and Nepal	Cross-sectional study	The rise of azithromycin-resistant typhoid in northern India highlights a broader issue in South Asia and emphasizes the urgent need for typhoid conjugate vaccines in the region.
Ullah I et al	2021	Pakistan	Review article	Mortality rates rise because of MDR typhoid in Pakistan. Treatment of XDR-typhoid has become challenging due to the irrational use of antibiotics. With the emergence of azithromycin-resistant strain, typhoid treatment has become untreatable. Rational use of azithromycin is essential.
Saleem Z et al	2023	Pakistan	Editorial	Self-medication is a significant concern in Pakistan. To manage risks, the WHO AWaRe categorizes antibiotics into ‘Access,’ ‘Watch,’ and ‘Reserve. ‘ Educating community pharmacists and their assistants on antibiotics and antimicrobial resistance (AMR) is crucial. Addressing rising AMR rates will remain a priority.
Hooda Y et al	2020	Niger, Malawi and Tanzania, Pakistan	Commentary	The rise of azithromycin resistance limits the benefits of mass drug administration. While azithromycin is a promising tool against childhood mortality, the lack of new drugs necessitates cautious consideration of the opportunity costs associated with mass administration.
Carey ME et al	2022	South-Asian Countries	Meta-analysis	XDR-typhoid is a common strain in Pakistan (70% of cases in 2020). Eight non-XDR genotypes show ceftriaxone resistance, and there’s a ciprofloxacin-resistant lineage in India. We can better track antibiotic-resistant Typhi and support typhoid conjugate vaccines (TCVs) through data sharing and collaboration.
Khan FZ et al	2018	Pakistan	Cross-sectional study	There is concern over MDR, fluoroquinolone-resistant, and azithromycin-resistant isolates of S. typhi. We recommend implementing antibiotic stewardship programs, promoting vaccination, and exploring resistance mechanisms to combat this trend.
Browne AJ et al	2024	Worldwide data	Comprehensive systematic review	The high prevalence of MDR, fluoroquinolone, and third-generation cephalosporin resistance in S Typhi and S Paratyphi A underscores the rising levels of antimicrobial resistance (AMR) to prevention. Urgent public health interventions are the solutions, including enhanced water, sanitation, hygiene, and typhoid vaccination.

## DISCUSSION

### Growing Threat of Azithromycin Resistance in S. Typhi in Endemic Regions:

Azithromycin is one of the few antibiotics still effective against the extensively drug-resistant strains of S. Typhi. However, the emergence of azithromycin-resistant strains is a highly Serious concern. All the included studies showed that typhoid fever is caused by an infection with Salmonella Typhi, which invades the intestinal mucosa and can spread systemically throughout the body. The increasing antibiotic resistance, including multi-drug-resistant (MDR) and extensively drug-resistant strains, has made treatment more complicated. Effective management of typhoid fever necessitates antibiotic stewardship, widespread vaccination, and significant improvements in sanitation. This study provides a comprehensive review of the epidemiology of typhoid, resistance patterns, and potential control strategies, specifically in Southeast Asia, concerning issues in endemic areas with high mortality and morbidity.^9^ Multiple studies from Pakistan have reported azithromycin resistance. Literature documented the emergence of azithromycin-resistant S. Typhi strains in Pakistan. Further exploration of genetics revealed a single point mutation in the acriflavine resistance B. (AcrB) gene in the resistant isolate with a minimum inhibitory concentration (MIC) of 12 μg/ml. In contrast, the MIC was observed to be 1-2 μg/ml in azithromycin-susceptible strains.^10^

Jabeen et al. discovered azithromycin resistance in 2% of the total isolates.^11^ Another study in Lahore, Pakistan, by Ahmad et al., found that 7.3% of XDR isolates had azithromycin resistance.^12^ A similar report mentioned that 2% of the isolates from CMH, Lahore, were resistant to azithromycin.^13^ A study from Agha Khan Hospital, Karachi, found 4.5% azithromycin treatment failure cases in extensively drug-resistant S. Typhi.^14^ Sajib et al. reported 38 cases of azithromycin-resistant Salmonella Typhi and Paratyphi A strains from 2016 to 2018 in Bangladesh. Genomic analysis detected single nucleotide polymorphism (SNP) in resistant isolates in the efflux pump AcrB (R717Q/L).^15^

Another study in Bangladesh by Ahsan et al. in 2019 documented resistance to azithromycin in 95% of the total test isolates. Azithromycin’s MIC values varied from 32 to 128 μg/ml. The erm(B) macrolide resistance gene was found in 62.5% of the resistant isolates.^16^ Duy et al. identified three azithromycin-resistant isolates in Nepal in 2019. All three isolates were found to be genetically identical and expressed single point mutation at codon 717 of the acrB gene (STY0519) that converts arginine to leucine.^17^

Carey et al. reported that seven S. Typhi organisms in Northern India possessed an R717Q mutation in the acrB gene. Moreover, six of these isolates had mutations in the gyrA (S83F and D87N) and parC (S80I) genes, which conferred resistance to ciprofloxacin. These modern isolates that were resistant to azithromycin and ciprofloxacin differed phylogenetically from one another as well as from those that had been reported from Pakistan, Bangladesh, and Nepal.^18^

### Unregulated Azithromycin Use and Rising Resistance in Pakistan:

Azithromycin resistance has increased explicitly in Pakistan in recent years. Widespread and uncontrolled use of azithromycin for the treatment of COVID-19 during 2020 and 2021 has significantly contributed to the emergence of resistance against azithromycin. Unfortunately, the uncertainty and unpredictability of COVID-19 disease perplexed physicians and led to the hit-and-trial use of multiple antibiotics. No set criteria were developed regarding antibiotic prescription. This was further worsened by aberrant compliance to complete the antibiotic course.^19^

Another reason for the up-roaring azithromycin resistance in Pakistan is the poor drug regulatory control and availability of azithromycin over the counter, without prescription. This has led to self-medication by the patients and the use of azithromycin for minor ailments such as pharyngitis and upper respiratory infections. Lack of culture and sensitivity before antibiotic prescription has become a routine and led to resistance against numerous effective drugs.^20^

Azithromycin resistance may limit the benefits of mass drug administration. The burning concern of antimicrobial resistance necessitates carefully considering the opportunity costs associated with mass administration.^21^ In the same respect, the XDR-typhoid strain in Pakistan was responsible for 70% of cases in 2020, and 8 non-XDR genotypes showed ceftriaxone resistance. The same study suggests that enhancing data sharing and typhoid conjugate vaccines (TCVs) will make better control of antibiotic-resistant Typhi possible.^22^

### Preventive Strategies to Combat Typhoid Resistance:

There are alarming issues with multidrug-resistant (MDR), fluoroquinolone-resistant, and azithromycin-resistant isolates of S. Typhi. This growing resistance complicates treatment and poses a significant public health threat. Addressing this challenge requires a multifaceted approach. Antibiotic stewardship programs can promote the prudent use of antibiotics and minimize the misuse that contributes to resistance.^23^

Additionally, urgent public health interventions are necessary to improve sanitation and access to clean water in affected regions. These measures can significantly reduce the transmission of S. Typhi. Vaccination also plays a critical role; increasing coverage can help prevent the spread of typhoid fever and reduce the disease burden. By combining these strategies, we can effectively control the emergence and spread of resistant strains and protect public health.^24^

### Future directions:

To address the issue of rising azithromycin resistance, immediate regulatory measures are needed, including preventing over-the-counter availability. Physicians should practice antibiotic stewardship by avoiding unnecessary prescriptions, and mass media campaigns should raise awareness about antibiotic misuse. In Pakistan, where typhoid fever cases are increasing, and treatment options are limited, controlling this resistance is critical. Disease prevention strategies, such as ensuring clean drinking water and promoting handwashing and hygiene, are essential. The typhoid conjugate vaccine is safe for infants and children, and the WHO has conducted mass vaccination campaigns since 2019. Overall, a multifaceted approach is necessary to reduce the disease burden and combat the emergence of antibiotic resistance.

### Limitations:

This systematic review has several limitations that merit attention. Firstly, it includes only studies published in English, which may introduce language bias and exclude relevant research in other languages. Secondly, the variability in methodology and study design among the included studies could impact the comparability of results. Additionally, the predominant focus on low-income and middle-income countries may limit the generalizability of the findings. Therefore, it is essential to recognize that the conclusions reflect a specific point in time and may not encompass the latest developments in healthcare practices and policies.

## CONCLUSION

Azithromycin resistance is rapidly emerging in Southeast Asia, posing a significant threat to treating typhoid fever if resistant strains become more widespread. To prevent a large-scale outbreak, it is essential to halt the irrational use of azithromycin. The rise of drug-resistant strains represents a serious challenge to treatment options. This resistance mainly stems from antibiotic misuse. Effective control requires improved sanitation and hygiene, widespread vaccination, prudent antibiotic use, and enhanced surveillance to track drug resistance. Addressing typhoid in Southeast Asia demands a comprehensive approach to combat this ongoing health threat.

### Author’s Contribution:

**SZ:** Initiated the literature review and analysis, synthesized the initial draft of a manuscript, and revised the draft.

**AS:** Conceptualized the idea, conceived the review, guided analysis and critically revised the manuscript.

## References

[1] Masuet-Aumatell C, Atouguia J (2021). Typhoid fever infection–Antibiotic resistance and vaccination strategies:A narrative review. Travel Med Infect Dis.

[2] Hancuh M, Walldorf J, Minta AA, Tevi-Benissan C, Christian KA, Nedelec Y (2023). Typhoid Fever Surveillance, Incidence Estimates, and Progress Toward Typhoid Conjugate Vaccine Introduction - Worldwide, 2018-2022. MMWR Morb Mortal Wkly Rep.

[3] Garrett DO, Longley AT, Aiemjoy K, Yousafzai MT, Hemlock C, Yu AT (2022). Incidence of typhoid and paratyphoid fever in Bangladesh, Nepal, and Pakistan:results of the Surveillance for Enteric Fever in Asia Project. Lancet Glob Health.

[4] Manesh A, Meltzer E, Jin C, Britto C, Deodhar D, Radha S (2021). Typhoid and paratyphoid fever:a clinical seminar. J Travel Med.

[5] Bhutta ZA (2006). Current concepts in the diagnosis and treatment of typhoid fever. BMJ.

[6] Butler T (2011). Treatment of typhoid fever in the 21st century:promises and shortcomings. Clin Microbiol Infect.

[7] Walker J, Chaguza C, Grubaugh ND, Carey M, Baker S, Khan K (2023). Assessing the global risk of typhoid outbreaks caused by extensively drug resistant Salmonella Typhi. Nat Commun.

[8] Qureshi S, Naveed AB, Yousafzai MT, Ahmad K, Ansari S, Lohana H (2020). Response of extensively drug resistant Salmonella Typhi to treatment with meropenem and azithromycin, in Pakistan. PLoS Negl Trop Dis.

[9] Jabeen K, Saleem S, Nizamuddin S, Arshad F, Jahan S, Hasnain F (2023). Reporting of azithromycin activity against clinical isolates of extensively drug-resistant Salmonella enterica Serovar Typhi. Am J Trop Med Hyg.

[10] Iqbal J, Dehraj IF, Carey ME, Dyson ZA, Garrett D, Seidman JC (2020). A race against time:reduced azithromycin susceptibility in Salmonella enterica serovar Typhi in Pakistan. MSphere.

[11] Jabeen K, Saleem S, Jahan S, Nizamudin S, Arshad F, Huma ZE (2023). Molecular characterization of extensively drug resistant Salmonella enterica Serovar Typhi clinical isolates from Lahore, Pakistan. Infect Drug Resist.

[12] Ahmad S, Tsagkaris C, Aborode AT, Haque MT, Khan SI, Khawaja UA (2021). A skeleton in the closet:the implications of COVID-19 on XDR strain of typhoid in Pakistan. Public Health Pract. (Oxf).

[13] Fida S, Mansoor H, Saif S, Iqbal J, Khan AQ (2021). Clinical Perspectives of Multiple and Extensively Drug-Resistant Typhoid;result from a tertiary care hospital from Pakistan. J. Infect. Dev. Ctries.

[14] Aziz S, Malik L (2018). Emergence of Multi-Resistant Enteric Infection in A Paediatric Unit of Karachi, Pakistan. J Pak Med Assoc.

[15] Sajib MS, Tanmoy AM, Hooda Y, Rahman H, Andrews JR, Garrett DO (2021). Tracking the emergence of azithromycin resistance in multiple genotypes of typhoidal salmonella. MBio.

[16] Ahsan S, Rahman S (2019). Azithromycin Resistance in Clinical Isolates of Salmonella enterica Serovars Typhi and Paratyphi in Bangladesh. MDR.

[17] Duy PT, Dongol S, Giri A, Nguyen To NT, Dan Thanh HN, Nhu Quynh NP (2020). The emergence of azithromycin-resistant Salmonella Typhi in Nepal. JAC Antimicrob Resist.

[18] Carey ME, Jain R, Yousuf M, Maes M, Dyson ZA, Thu TN (2021). Spontaneous Emergence of Azithromycin Resistance in Independent Lineages of Salmonella Typhi in Northern India. Clin Infect Dis.

[19] Ullah I, Khan KS, Mehmood Q, Tahir MJ, Malik MI, Ahmed A (2021). Irrational use of azithromycin in typhoid endemic areas:A challenge on multidrug-resistant typhoid treatment. Trends Infect Glob Health.

[20] Saleem Z, Sono TM, Godman B (2023). Concerns with current Drug Laws regarding the purchasing antibiotics without a prescription in Pakistan;ways forward to assist the national action plan. Expert Rev Anti Infect Ther.

[21] Hooda Y, Tanmoy AM, Sajib MS, Saha S (2020). Mass azithromycin administration:considerations in an increasingly resistant world. BMJ Global Health.

[22] Carey ME, Dyson ZA, Ingle DJ, Amir A, Aworh MK, Chattaway MA, Chew KL, Crump JA, Feasey NA, Howden BP, Keddy KH (2023). Global Typhoid Genomics Consortium Group Authorship. Global diversity and antimicrobial resistance of typhoid fever pathogens:Insights from a meta-analysis of 13,000 Salmonella Typhi genomes. Elife.

[23] Khan FZ, Baig S, Zameer S, Sharafat S (2018). Emerging Trends of Antibiotic Resistance pattern of Salmonella Typhi. Int J Pathol.

[24] Browne AJ, Chipeta MG, Fell FJ, Haines-Woodhouse G, Hamadani BH, Kumaran EA (2024). Estimating the subnational prevalence of antimicrobial resistant Salmonella enterica serovars Typhi and Paratyphi A infections in 75 endemic countries, 1990–2019:a modelling study. Lancet Global Health.

